# Progression of coronary artery calcification in chronic kidney disease: nonlinear associations with serum magnesium and parathyroid hormone

**DOI:** 10.3389/fmed.2026.1910302

**Published:** 2026-09-15

**Authors:** Yutong Chen, Wenhao Qiu, Yinglan Shu, Lifeng Lu, Yindeng Luo

**Affiliations:** 1 Department of Radiology, The Second Affiliated Hospital of Chongqing Medical University, Chongqing, China; 2 School of Computer Science and Technology, Chongqing University of Posts and Telecommunications, Chongqing, China

**Keywords:** chronic kidney disease, coronary artery calcification (CAC), dose-response relationship, parathyroid hormone, progression trajectory, serum magnesium (Mg)

## Abstract

**Introduction:**

Coronary artery calcification (CAC) progresses rapidly in chronic kidney disease (CKD), but its trajectories across stages and nonlinear relationships with serum magnesium and parathyroid hormone (PTH) remain unclear.

**Methods:**

We evaluated 800 CKD patients stratified into four groups (stages 1–2, 3, 4–5, and dialysis) over a median follow-up of 20.5 months using serial chest CT scans. Linear mixed-effects models, restricted cubic splines, and LASSO regression were applied to analyze trajectories, nonlinear relationships, and construct a predictive model.

**Results:**

The annualized rate of CAC progression increased significantly with advancing CKD stages (8.5%, 13.2%, 22.8%, and 29.5%/year, respectively; *P* < 0.001). Serum magnesium exhibited an L-shaped nonlinear relationship with rapid CAC progression (inflection point: 0.82 mmol/L), while PTH showed a J-shaped relationship (inflection point: 120 pg/mL). Uric acid was linearly and positively correlated. A 7-factor LASSO-derived prognostic model demonstrated robust performance, yielding a C-statistic of 0.802 (95% CI: 0.772-0.832) and good calibration.

**Conclusion:**

In conclusion, CAC progression accelerates as renal function declines, peaking in dialysis patients. Low magnesium (<0.82 mmol/L) and high PTH (120 pg/mL) are key nonlinear risk factors. The resulting predictive model provides clinical utility for stratified cardiovascular risk management.

## Graphical Abstract

**Figure:**
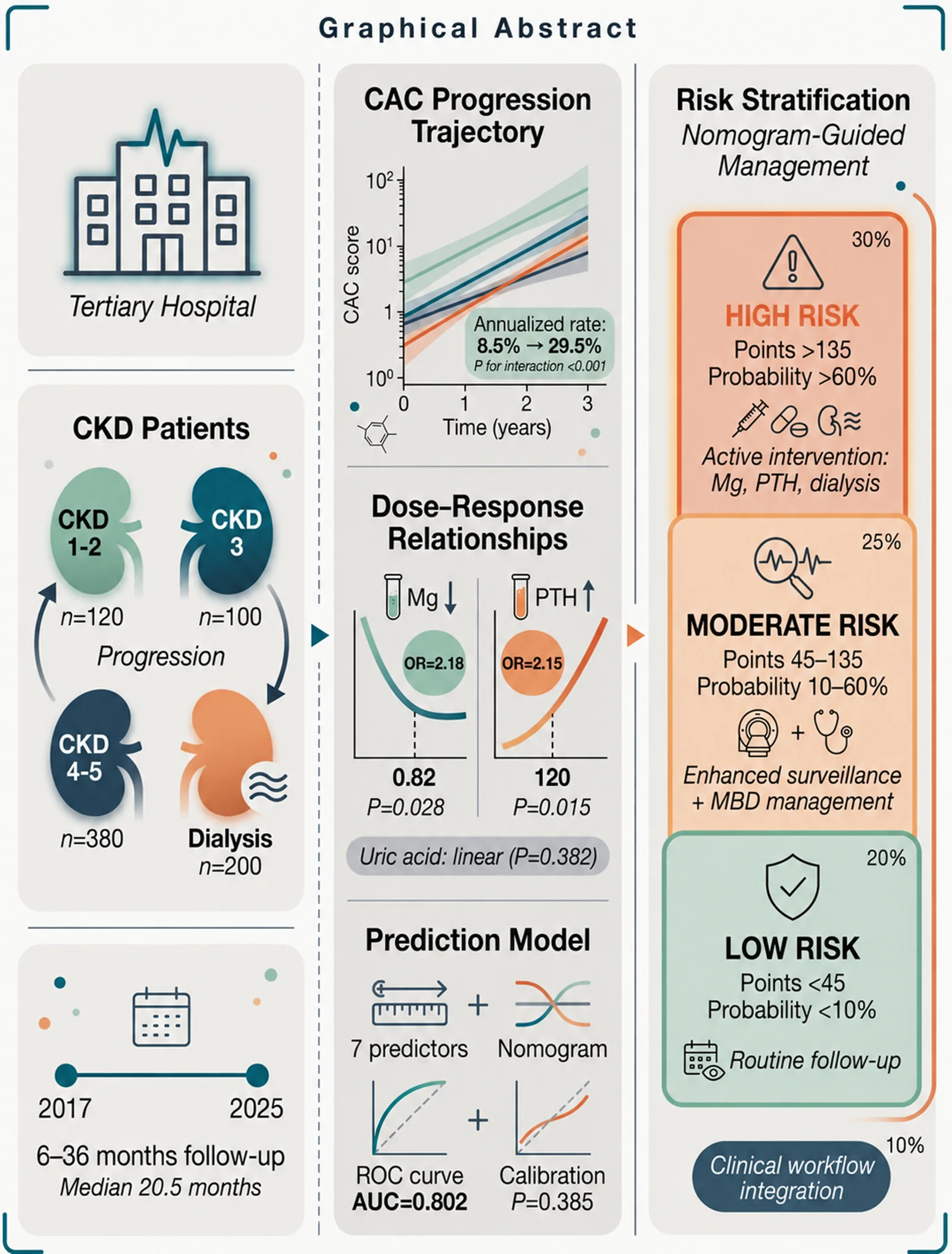

## Introduction

Chronic kidney disease (CKD) is a major global public health issue. With an estimated worldwide prevalence of 9.5%, CKD affects more than 800 million adults, and its burden continues to increase with population aging and the growing prevalence of diabetes and hypertension (1). Patients with CKD have a markedly elevated risk of cardiovascular disease, with cardiovascular mortality rates 5–10 times higher than those in the general population. Traditional cardiovascular risk factors do not fully explain this excess risk, suggesting that uremia-specific mechanisms have an important role in cardiovascular complications of CKD (2, 3).

Coronary artery calcification (CAC) is a core pathological feature of cardiovascular disease in CKD. In this population, vascular calcification is not simply a passive degenerative process; rather, it is actively regulated and is driven in part by osteogenic transformation of vascular smooth muscle cells (4). CAC scores are positively associated with future cardiovascular events and all-cause mortality in patients with CKD, and even asymptomatic patients with high CAC scores have a poor prognosis (5, 6). CAC in CKD also tends to progress continuously, with substantial heterogeneity across individuals. Recent studies suggest that rapid progression of calcification may predict cardiovascular events and progression to end-stage kidney disease more effectively than a single CAC measurement (7, 8). Identifying patients at high risk for rapid CAC progression and clarifying modifiable predictors are therefore important for cardiovascular risk management in CKD.

Disordered calcium-phosphorus metabolism is a central driver of vascular calcification in CKD. Serum magnesium, a natural calcium antagonist, may inhibit calcification through several mechanisms, including suppression of hydroxyapatite crystal formation and regulation of osteogenic differentiation in vascular smooth muscle cells (9). Clinical studies have linked hypomagnesemia to greater vascular calcification and disease progression in CKD (10). However, most existing evidence is cross-sectional or based on small cohorts, and whether serum magnesium has a nonlinear dose-response association with CAC progression remains unclear. Parathyroid hormone (PTH) is another key regulator of mineral metabolism, and PTH levels rise as kidney function declines. Evidence on PTH and CAC progression has been inconsistent: some studies suggest that high PTH promotes vascular calcification, whereas others indicate that both very low and very high PTH levels are associated with poor outcomes, suggesting a U-shaped relationship (11, 12). The role of serum uric acid, a common marker of metabolic disturbance, also remains uncertain because many studies have not fully accounted for kidney function and mineral metabolism markers (13).

Several gaps remain. First, longitudinal repeated-measure data have not been used extensively to describe the natural trajectory of CAC across CKD stages, including both non-dialysis and dialysis populations. Second, prior studies have often evaluated magnesium, PTH, and uric acid separately, leaving their independent and potentially nonlinear associations with CAC progression incompletely characterized. Third, clinically practical tools that integrate these markers to identify patients at high risk for rapid CAC progression are limited. Therefore, this study used longitudinal cohort data from 800 patients with CKD to characterize CAC trajectories across CKD stages, evaluate the independent and nonlinear associations of baseline serum magnesium, PTH, and uric acid with rapid CAC progression, and internally validate a risk prediction nomogram for stratified cardiovascular risk assessment.

## Materials and methods

### Study design and population

This retrospective longitudinal cohort study included patients with CKD who underwent regular follow-up at the Nephrology Department and Hemodialysis Center of the Second Affiliated Hospital of Chongqing Medical University between January 2017 and January 2025.Serial chest CT examinations were clinically indicated rather than performed as part of a universal screening policy for patients with CKD. The clinical indications included the evaluation of suspected cardiovascular or cardiopulmonary disease, follow-up of known cardiopulmonary conditions, and preoperative assessment. The decision to perform and repeat CT examinations, including the timing of follow-up imaging, was made by the treating physicians according to clinical need and was not specified by the study protocol. During the study period, 874 CKD patients with serial CT scans were initially screened. Inclusion criteria were: (1) age ≥18 years; (2) diagnosis of CKD, defined as persistent structural or functional kidney abnormalities for more than 3 months; (3) at least two chest CT scans for CAC scoring separated by ≥6 months; and (4) available baseline serum magnesium, PTH, uric acid, and routine biochemical measurements. After applying the exclusion criteria—(1) acute cardiovascular events at baseline (*n* = 12);(2) primary hyperparathyroidism (*n* = 14);(3) adjustment of long-term magnesium supplements or phosphate binders within 3 months before baseline (*n* = 40)(to ensure the baseline stability of mineral metabolism markers and avoid acute medication-induced fluctuations);(4) active malignancy or expected survival <1 year (*n* = 6); (5) pregnancy or lactation (*n* = 2)—a final cohort of 800 patients (482 males; mean age, 59.2 ± 12.8 years) was included in the analysis. The Ethics Committee of the Second Affiliated Hospital of Chongqing Medical University approved the study and waived the requirement for informed consent because of the retrospective design. The study was reported in accordance with the STROBE guideline for observational research. The study design is summarized in Figure 1.

**Figure 1:**
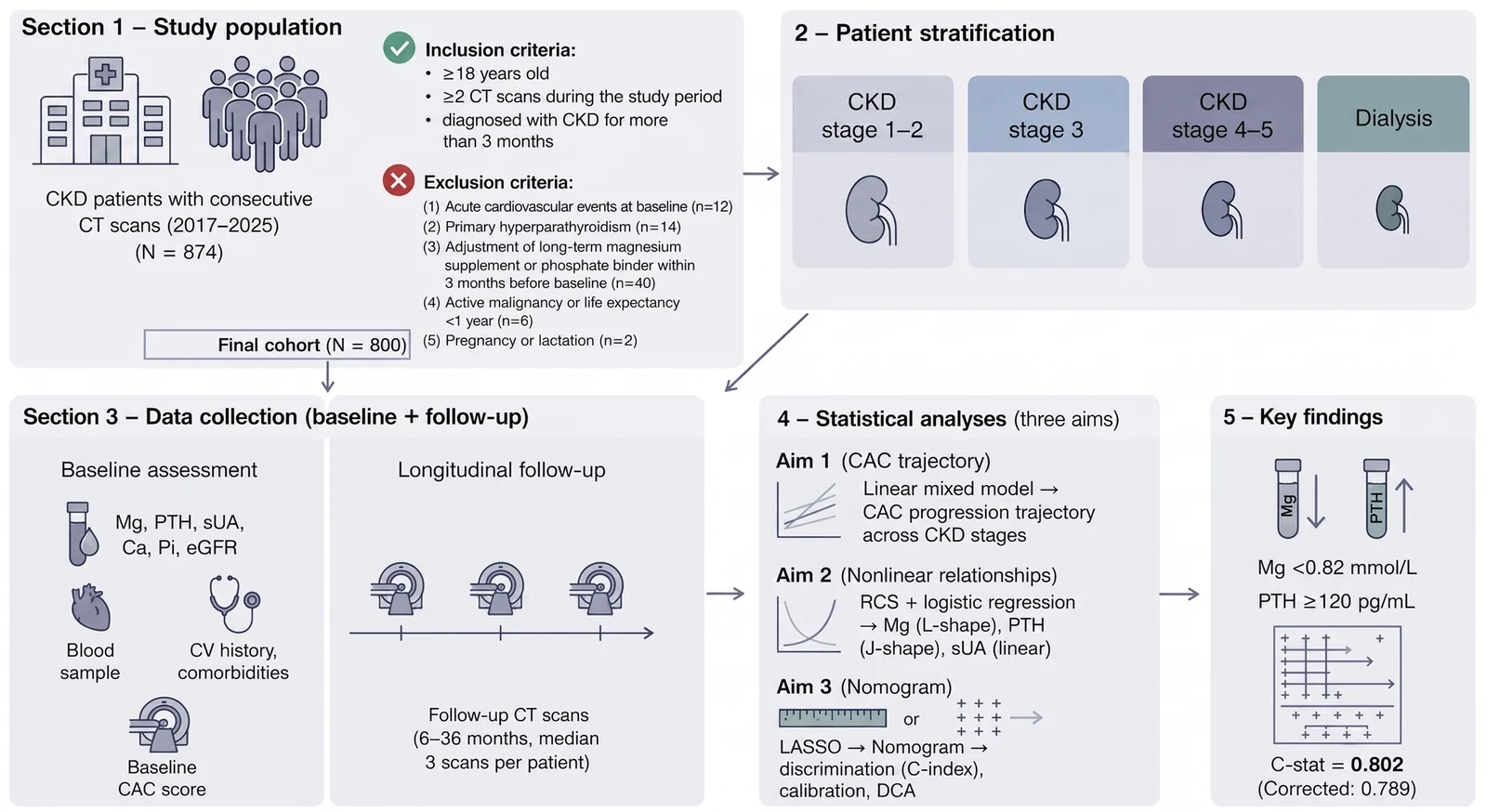
Schematic diagram of the study design and analysis workflow. This figure presents the overall study design. From left to right: (1) study population, including patients with CKD enrolled from 2017 to 2025; (2) patient stratification into CKD stages 1-2, stage 3, stages 4-5, and dialysis groups, with dot plots indicating group size; (3) data collection at baseline, including blood biochemistry, medical history, and CT scan, and follow-up, including serial CT scans between 6 and 36 months; (4) statistical analyses, including trajectory analysis, dose-response analysis, and nomogram construction; and (5) key findings, including serum magnesium <0.82 mmol/L, PTH ≥120 pg/mL, and a model C-statistic of approximately 0.80.

### Data collection

Data were extracted from the electronic medical record system into a standardized database. Baseline was defined as the date of the initial CT scan and paired blood sampling. Collected variables included sociodemographic characteristics, lifestyle factors, medical history, and CKD stage, categorized according to baseline estimated glomerular filtration rate (eGFR), with dialysis status recorded separately. Fasting venous blood samples were analyzed by the central laboratory. Routine biochemical variables, including serum calcium, phosphorus, magnesium, and uric acid, were quantitatively measured using an automated clinical chemistry analyzer (Beckman Coulter AU5800, USA). Serum PTH was measured by electrochemiluminescence immunoassay on an automated immunology analyzer (Roche Cobas e601, Switzerland). eGFR was calculated using the MDRD formula. CAC scores were quantified on multislice spiral CT using the Agatston scoring method and included the right coronary artery, left main coronary artery, left anterior descending artery, and left circumflex artery. For follow-up scans, intervals from baseline and between consecutive scans were calculated to determine annualized calcification progression.

### Outcome measures and definitions

The primary outcome was rapid CAC progression, defined as an absolute annualized increase in CAC score of ≥100 Agatston units or a relative increase of ≥10%, with baseline scores ≤100 used as the denominator. Secondary analyses used the continuous annualized rate of change to assess robustness. CKD staging followed the 2024 KDIGO clinical practice guideline: stage G1, ≥90 mL/min/1.73 m^2^; G2, 60–89 mL/min/1.73 m^2^; G3a, 45–59 mL/min/1.73 m^2^; G3b, 30–44 mL/min/1.73 m^2^; G4, 15–29 mL/min/1.73 m^2^; and G5, <15 mL/min/1.73 m^2^. Magnesium, PTH, and uric acid were analyzed both continuously and categorically according to nonlinear dose-response percentiles.

### Statistical analysis

Continuous variables were assessed for normality using the Kolmogorov–Smirnov test. Normally distributed variables are presented as mean ± standard deviation and were compared using analysis of variance. Non-normally distributed variables are presented as median and interquartile range (IQR) and were compared using the Kruskal–Wallis H test. Categorical variables are presented as frequencies and percentages and were compared using the chi-square test.

A linear mixed-effects model was used to characterize total CAC trajectories across CKD stages. CAC scores were log-transformed as ln(CAC score + 1) to address right skewness. Follow-up time was treated as a continuous fixed effect, and patient-specific random intercepts and slopes were included. The model was adjusted for age, sex, and baseline cardiovascular history and included fixed effects for time, CKD stage, and the time-by-stage interaction.

Restricted cubic splines combined with multivariable logistic regression were used to evaluate independent and nonlinear associations of baseline magnesium, PTH, and uric acid with rapid CAC progression. Spline knots were placed at the 5th, 35th, 65th, and 95th percentiles. Likelihood ratio tests were used to assess nonlinearity. After nonlinear thresholds were identified by two-segment grid search, continuous exposures were converted into clinically interpretable categories to calculate adjusted odds ratios (ORs). Models were adjusted for demographic factors, clinical history, and laboratory covariates. Bonferroni correction was applied for multiple comparisons (*P* < 0.017).

Least absolute shrinkage and selection operator (LASSO) regression was used to select variables for the risk prediction nomogram from 34 baseline candidates. The L1 penalty shrank coefficients of less informative variables toward zero. Ten-fold cross-validation was used to identify the optimal penalization parameter (lambda.1se). Selected variables were then entered into a multivariable logistic regression model to construct the final nomogram.

Model discrimination was evaluated using the C-statistic. The Youden index was used to determine the optimal cutoff for sensitivity and specificity. Calibration was assessed using 1,000 bootstrap resamples and the Hosmer-Lemeshow test. Clinical utility was evaluated using decision curve analysis. Statistical analyses were performed in R version 4.3.1 using the lme4, rms, glmnet, pROC, and rmda packages. Two-sided *P*-values <0.05 were considered statistically significant.

## Results

### Baseline characteristics of the study population

The study included 800 patients with CKD, including 482 men (60.3%) and 318 women (39.7%), with a mean age of 59.2 ± 12.8 years. Based on baseline eGFR and dialysis status, 120 patients (15.0%) were in CKD stages 1–2, 100 (12.5%) were in stage 3, 380 (47.5%) were in stages 4–5, and 200 (25.0%) were receiving dialysis.

Follow-up ranged from 6 to 36 months, with a median follow-up of 20.5 months (IQR, 11.0–32.0 months). Overall, 112 patients (14.0%) were followed for less than 12 months, 328 (41.0%) for 12–24 months, and 360 (45.0%) for 24–36 months. The median number of CT scans per patient was 3 (range, 2–5), and the median interval between consecutive CT scans was 9.8 months (IQR, 6.5–13.2 months).

At baseline, 584 patients (73.0%) had hypertension, 312 (39.0%) had diabetes, 356 (44.5%) had hyperlipidemia, 176 (22.0%) had a personal history of cardiovascular disease, and 248 (31.0%) had a family history of cardiovascular disease. There were 240 smokers (30.0%) and 192 alcohol drinkers (24.0%).

The median serum magnesium level was 0.84 mmol/L (IQR, 0.75–0.92 mmol/L), and 168 patients (21.0%) had hypomagnesemia (<0.75 mmol/L). The median PTH level was 85.6 pg/mL (IQR, 42.5–168.3 pg/mL). The median serum uric acid level was 428 μmol/L (IQR, 356–508 μmol/L). The median baseline CAC score was 98.6 (IQR, 18.5–386.5), and 168 patients (21.0%) had a baseline CAC score of 0.

Across worsening CKD stages, age increased slightly, the prevalence of hypertension and diabetes increased, serum magnesium levels tended to decrease, and PTH and uric acid levels tended to increase. Baseline CAC scores also increased across CKD stages (Figure 2 and Table 1).

**Figure 2:**
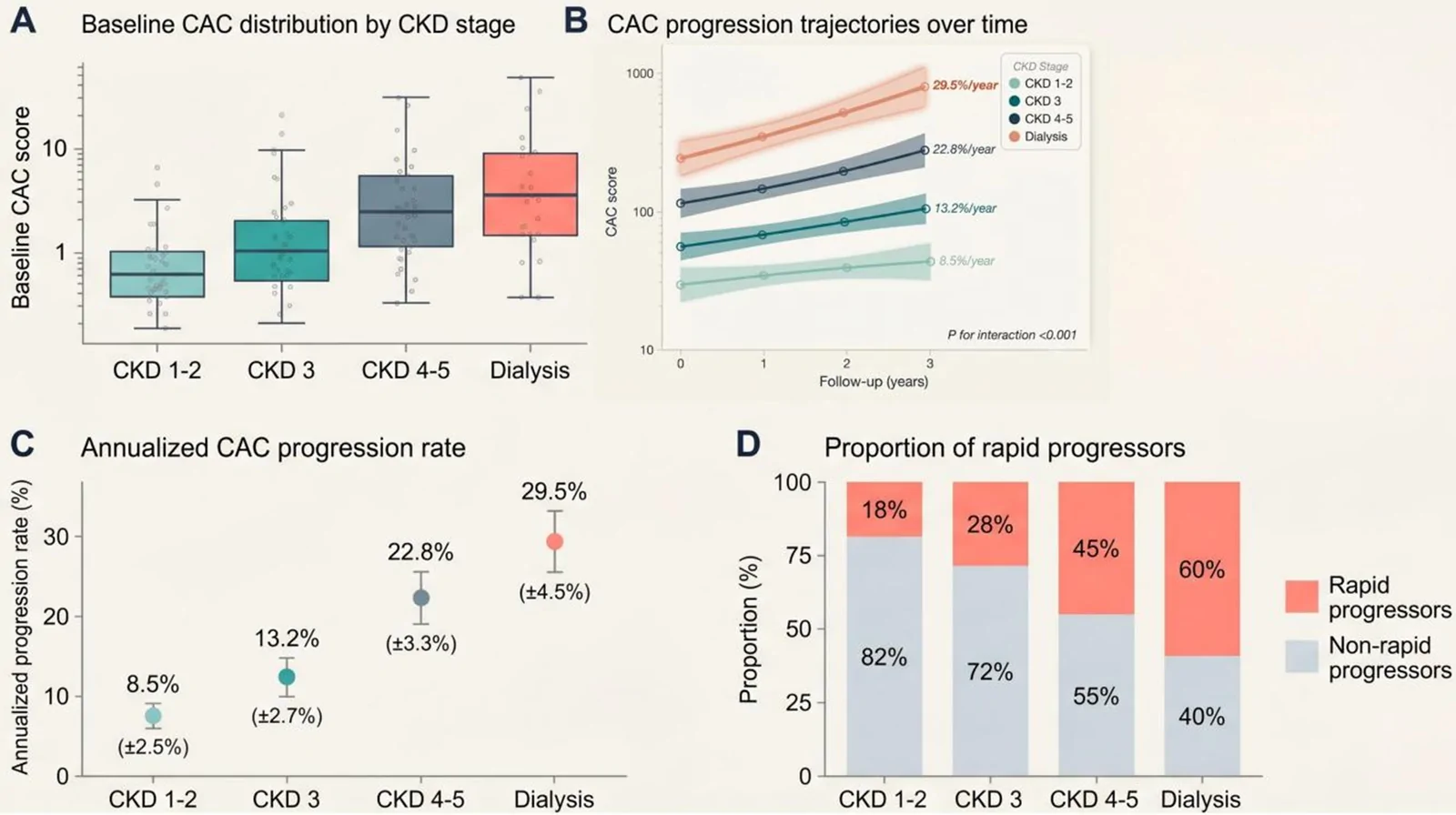
Trajectories of coronary artery calcification progression across CKD stages. **(A)** Distribution of baseline CAC scores across CKD stages. **(B)** CAC progression trajectories over time by CKD stage, estimated using a linear mixed-effects model. **(C)** Annualized CAC progression rates across CKD stages. Data are presented as medians and 95% confidence intervals. **(D)** Proportion of patients with rapid CAC progression across CKD stages. Rapid progression was defined as an absolute annual increase in CAC score of ≥100 Agatston units or a relative increase of ≥10%. CAC, coronary artery calcification; CKD, chronic kidney disease.

**Table 1:** Baseline characteristics of the study population.

Characteristic	Overall (*N* = 800)	CKD Stages 1–2 (*n* = 120)	CKD Stage 3 (*n* = 100)	CKD Stages 4–5 (*n* = 380)	Dialysis (*n* = 200)	*P*-value
Demographic characteristics						
Age (years, mean ± SD)	59.2 ± 12.8	54.5 ± 12.6	56.8 ± 12.4	60.2 ± 12.6	60.8 ± 11.8	0.002
Male, *n* (%)	482 (60.3)	68 (56.7)	58 (58.0)	232 (61.1)	124 (62.0)	0.785
Lifestyle						
Smoking, *n* (%)	240 (30.0)	34 (28.3)	28 (28.0)	114 (30.0)	64 (32.0)	0.562
Alcohol consumption, *n* (%)	192 (24.0)	30 (25.0)	22 (22.0)	90 (23.7)	50 (25.0)	0.852
Medical history						
Hypertension, *n* (%)	584 (73.0)	76 (63.3)	68 (68.0)	280 (73.7)	160 (80.0)	0.042
Diabetes, *n* (%)	312 (39.0)	36 (30.0)	34 (34.0)	152 (40.0)	90 (45.0)	0.045
Hyperlipidemia, *n* (%)	356 (44.5)	46 (38.3)	42 (42.0)	172 (45.3)	96 (48.0)	0.386
Personal history of cardiovascular disease, *n* (%)	176 (22.0)	18 (15.0)	20 (20.0)	86 (22.6)	52 (26.0)	0.125
Family history of cardiovascular disease, *n* (%)	248 (31.0)	38 (31.7)	30 (30.0)	116 (30.5)	64 (32.0)	0.892
Laboratory parameters						
eGFR (mL/min/1.73 m^2^, median, IQR)	22.5 (12.8–42.6)	86.5 (72.5–102.5)	48.5 (40.5–56.5)	18.5 (12.5–25.5)	NA	<0.001
Serum magnesium (mmol/L, median, IQR)	0.84 (0.75–0.92)	0.92 (0.86–0.98)	0.89 (0.83–0.95)	0.83 (0.75–0.90)	0.80 (0.72–0.88)	<0.001
Hypomagnesemia (<0.75), *n* (%)	168 (21.0)	14 (11.7)	16 (16.0)	86 (22.6)	52 (26.0)	0.008
PTH (pg/mL, median, IQR)	85.6 (42.5–168.3)	42.5 (32.5–58.6)	58.6 (42.5–85.6)	102.5 (58.6–186.5)	215.6 (145.6–356.8)	<0.001
Serum uric acid (*μ*mol/L, median, IQR)	428 (356–508)	368 (325–435)	398 (342–462)	438 (365–512)	462 (385–535)	<0.001
Serum calcium (mmol/L, mean ± SD)	2.27 ± 0.19	2.31 ± 0.13	2.30 ± 0.15	2.26 ± 0.18	2.23 ± 0.24	0.056
Serum phosphorus (mmol/L, mean ± SD)	1.38 ± 0.36	1.18 ± 0.24	1.25 ± 0.28	1.42 ± 0.36	1.52 ± 0.42	<0.001
Albumin (g/L, mean ± SD)	38.2 ± 5.4	39.8 ± 4.6	39.0 ± 5.0	38.0 ± 5.4	36.8 ± 5.2	0.015
Coronary artery calcification						
Baseline CAC score (median, IQR)	98.6 (18.5–386.5)	32.5 (6.5–128.5)	68.5 (12.5–256.5)	128.5 (32.5–428.5)	268.5 (95.6–512.5)	<0.001
CAC score = 0, *n* (%)	168 (21.0)	52 (43.3)	26 (26.0)	68 (17.9)	22 (11.0)	<0.001
Follow-up duration (months, median, IQR)	20.5 (11.0–32.0)	24.0 (14.0–35.0)	22.5 (13.0–34.0)	20.0 (10.5–31.0)	18.5 (10.0–30.0)	0.086

CAC, coronary artery calcification; CKD, chronic kidney disease; eGFR, estimated glomerular filtration rate; IQR, interquartile range; NA, not applicable; PTH, parathyroid hormone; SD, standard deviation.

### Progression trajectories of coronary artery calcification

During follow-up, 2,320 coronary CT scans were performed, with an average of 2.9 scans per patient. In the overall cohort, the median CAC score increased from 98.6 at baseline to 156.2 at the last follow-up (IQR, 32.5–512.6), with a median annualized rate of change of 18.5% per year (IQR, 5.6%–42.8% per year). According to the predefined criteria, 348 patients (43.5%) had rapid CAC progression.

In the linear mixed-effects model, follow-up duration was positively associated with CAC score (*β* = 0.168; 95% CI, 0.138–0.198; *P* < 0.001), indicating a sustained increase over time. CAC trajectories differed significantly across CKD stages (time-by-stage interaction, *P* < 0.001). The annualized rate of change was lowest in CKD stages 1–2 (8.5% per year; 95% CI, 6.0%–11.0%), intermediate in CKD stage 3 (13.2% per year; 95% CI, 10.5%–15.9%), higher in non-dialysis CKD stages 4–5 (22.8% per year; 95% CI, 19.5%–26.1%), and highest in dialysis patients (29.5% per year; 95% CI, 25.0%–34.0%). These differences remained statistically significant after adjustment for age, sex, and baseline cardiovascular history.

The high annualized progression rates in CKD stages 4–5 and dialysis patients suggest that vascular calcification may accelerate as kidney function approaches failure. Among the 112 patients followed for less than 12 months, the median annualized CAC progression rate was 20.2% per year, higher than the 16.8% per year observed among patients followed for ≥24 months, suggesting that early rapid progression may be clinically relevant (Figure 2 and Table 2).

**Table 2:** Predicted coronary artery calcium scores by CKD stage estimated using a linear mixed-effects model.

Follow-up Duration (Years)	CKD Stages 1–2 (*n* = 120)	CKD Stage 3 (*n* = 100)	CKD Stages 4–5 (*n* = 380)	Dialysis (*n* = 200)
0	35.6 (24.5–51.8)	72.5 (52.6–99.8)	132.5 (105.6–166.2)	272.5 (225.6–329.2)
0.5	37.2 (25.8–53.6)	77.2 (56.2–106.0)	148.2 (118.5–185.5)	312.8 (258.5–378.5)
1	38.8 (27.2–55.2)	82.2 (60.2–112.5)	165.8 (132.8–207.5)	359.5 (296.8–435.2)
1.5	40.5 (28.6–57.2)	87.5 (64.5–119.2)	185.5 (148.6–232.2)	413.5 (341.2–500.2)
2	42.3 (30.2–59.5)	93.2 (68.8–126.5)	207.8 (166.5–260.2)	475.8 (392.5–575.2)
2.5	44.2 (31.8–62.0)	99.2 (73.5–134.2)	232.5 (186.8–291.5)	547.2 (451.2–661.2)
3	46.2 (33.6–64.8)	105.6 (78.5–142.5)	260.5 (209.5–326.5)	629.5 (518.5–760.2)

Data represent median CAC scores and 95% confidence intervals estimated using a linear mixed-effects model adjusted for age, sex, and baseline cardiovascular history.

### Associations of Serum magnesium, PTH, and uric acid With rapid CAC progression

In univariate logistic regression, baseline serum magnesium was inversely associated with rapid CAC progression (OR=0.35; 95% CI, 0.20–0.62; *P* < 0.001). Serum PTH was positively associated with rapid CAC progression (OR=1.75; 95% CI, 1.48–2.08; *P* < 0.001 per 50 pg/mL increase), as was serum uric acid (OR=1.20; 95% CI, 1.06–1.36; *P* = 0.005 per 50 μmol/L increase).

Restricted cubic spline analysis showed a nonlinear association between serum magnesium and rapid CAC progression (nonlinearity test *P* = 0.028), with an approximately L-shaped curve. When serum magnesium was below 0.82 mmol/L, the risk of rapid CAC progression increased as magnesium decreased; above 0.82 mmol/L, the decline in risk tended to plateau. The optimal clinical cutoff based on inflection point analysis was 0.82 mmol/L.

PTH also showed a nonlinear association with rapid CAC progression (nonlinearity test *P* = 0.015), with a J-shaped curve. Below 120 pg/mL, the increase in risk was gradual; above 120 pg/mL, the risk of rapid CAC progression rose more steeply. The optimal clinical cutoff for PTH was 120 pg/mL.

No significant nonlinear association was observed between serum uric acid and rapid CAC progression (nonlinearity test *P* = 0.382); the association was approximately linear and positive (Figure 3 and Table 3).

**Figure 3:**
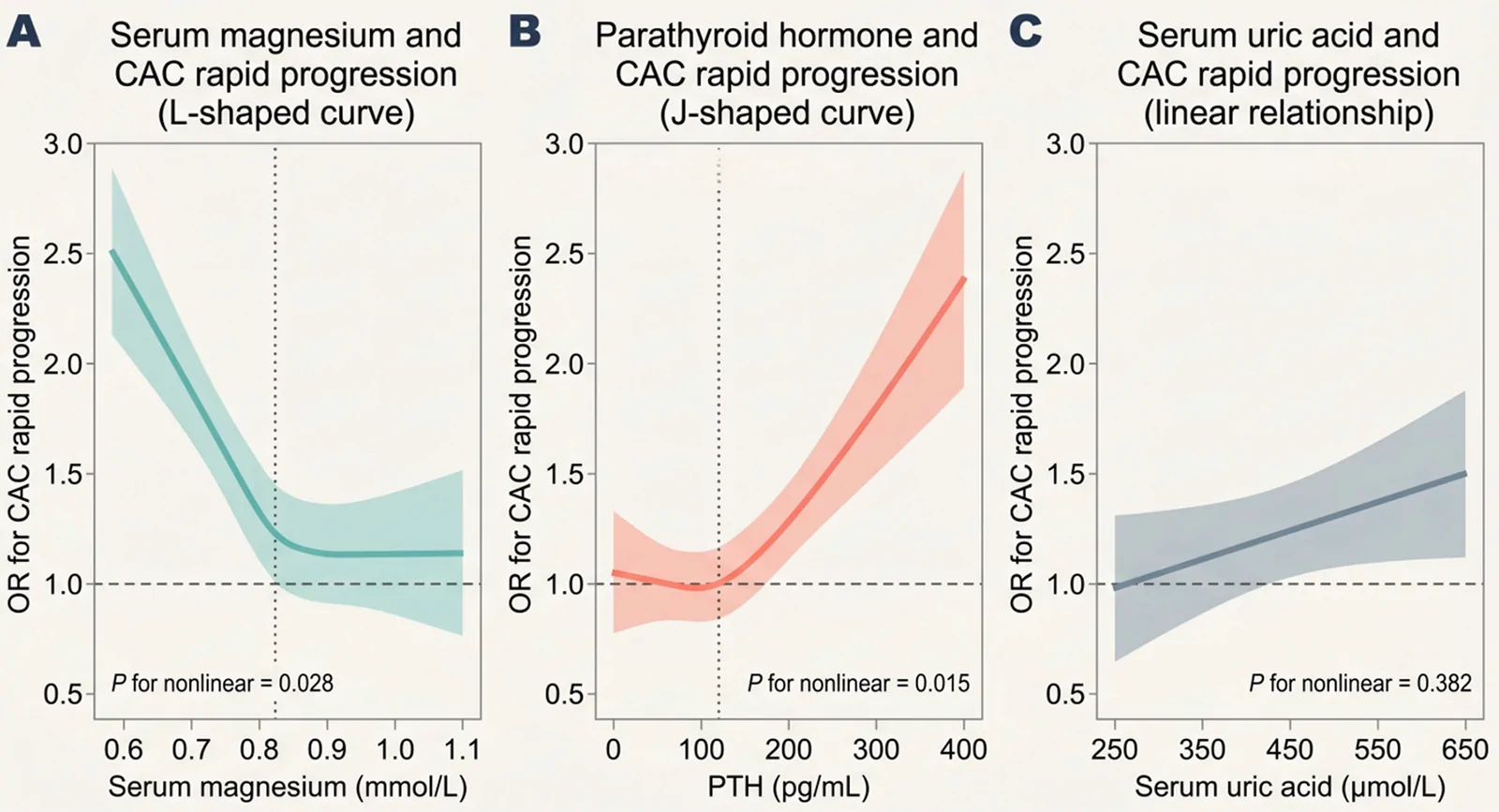
Dose-response relationships of serum magnesium, parathyroid hormone, and uric acid with rapid coronary artery calcification progression. **(A)** Restricted cubic spline curve for serum magnesium and rapid CAC progression. **(B)** Restricted cubic spline curve for PTH and rapid CAC progression. **(C)** Restricted cubic spline curve for serum uric acid and rapid CAC progression. Shaded areas represent 95% confidence intervals; the horizontal dashed line indicates the reference line (OR=1.0). CAC, coronary artery calcification; OR, odds ratio; PTH, parathyroid hormone.

**Table 3:** Multivariable logistic regression analysis of serum magnesium, PTH, uric acid, and rapid coronary artery calcification progression.

Variables	Univariate Analysis		Multivariate Analysis	
	OR (95% CI)	*P*-value	OR (95% CI)	*P*-value
Serum magnesium				
≥0.82 mmol/L (reference)	1.00		1.00	
<0.82 mmol/L	2.52 (1.88–3.38)	<0.001	2.18 (1.60–2.98)	<0.001
Parathyroid hormone				
<120 pg/mL (reference)	1.00		1.00	
≥120 pg/mL	2.45 (1.85–3.25)	<0.001	2.15 (1.58–2.92)	<0.001
Serum uric acid (per 50 μmol/L increase)	1.20 (1.06–1.36)	0.005	1.14 (1.00–1.30)	0.048

The multivariable model was adjusted for age, sex, diabetes, hypertension, baseline eGFR, dialysis status, serum calcium, serum phosphorus, and albumin.

**Table 4:** Multivariable logistic regression model for predicting rapid coronary artery calcification progression.

Predictor	*β* Coefficient	OR (95% CI)	*P*-value	Score (points)
Age (per 10-year increase)	0.292	1.34 (1.16–1.55)	<0.001	12
Baseline CAC score (per 100-unit increase)	0.392	1.48 (1.33–1.65)	<0.001	16
Serum magnesium <0.82 mmol/L	0.845	2.33 (1.70–3.20)	<0.001	35
PTH ≥ 120 pg/mL	0.778	2.18 (1.60–2.96)	<0.001	32
Diabetes (Yes)	0.538	1.71 (1.30–2.26)	<0.001	22
On dialysis (Yes)	0.625	1.87 (1.38–2.54)	<0.001	26
eGFR (per 10 mL/min/1.73 m^2^ increase)	−0.142	0.87 (0.79–0.96)	0.004	−6

Score values correspond to the nomogram points assigned to each predictor. The total score is the sum of component points and maps to the predicted probability of rapid CAC progression.

Serum calcium and phosphorus were included *a priori* as adjustment covariates in the multivariable logistic regression models. After adjustment for serum calcium, serum phosphorus, age, sex, diabetes, hypertension, baseline eGFR, dialysis status, and albumin, serum magnesium <0.82 mmol/L remained independently associated with rapid CAC progression (OR=2.18; 95% CI, 1.60–2.98; *P* < 0.001). PTH ≥120 pg/mL also remained independently associated with rapid CAC progression (OR=2.15; 95% CI, 1.58–2.92; *P* < 0.001). Each 50 μmol/L increase in serum uric acid was associated with a 14% higher risk of rapid CAC progression (OR=1.14; 95% CI, 1.00–1.30; *P* = 0.048). When magnesium and PTH were included simultaneously, both associations remained stable, and no significant interaction was observed (interaction *P* = 0.298).

Subgroup analysis showed that the association between serum magnesium and rapid CAC progression was broadly consistent across CKD stages. The association with PTH was stronger in patients with CKD stages 4–5 and in those receiving dialysis (OR=2.62; 95% CI, 1.80–3.82), whereas it did not reach statistical significance in patients with CKD stages 1–3 (OR=1.42; 95% CI, 0.92–2.18). These findings suggest that PTH may become a more prominent marker of calcification progression in advanced kidney dysfunction.

### Nomogram construction and performance evaluation

LASSO regression selected seven predictors of rapid CAC progression from 34 candidate variables, which included serum calcium and phosphorus: age (coefficient = 0.078), baseline CAC score (coefficient = 0.292), serum magnesium <0.82 mmol/L (coefficient = 0.305), PTH ≥120 pg/mL (coefficient = 0.282), diabetes (coefficient = 0.202), dialysis status (coefficient = 0.238), and baseline eGFR (coefficient = −0.045 per 10 mL/min/1.73 m^2^ increase). Serum uric acid, calcium, and phosphorus were not retained at the selected penalty parameter. The selected variables were subsequently entered into a multivariable logistic regression model to construct the final nomogram (Table 4). In the nomogram, serum magnesium <0.82 mmol/L corresponded to 35 points, PTH ≥120 pg/mL to 32 points, dialysis status to 26 points, and diabetes to 22 points. Each 100-unit increase in baseline CAC score contributed 16 points, each 10-year increase in age contributed 12 points, and each 10 mL/min/1.73 m^2^ increase in eGFR deducted 6 points. The total score ranged from 0 to 180 points and mapped to predicted probabilities of rapid CAC progression from <10% (total score <45) to >60% (total score >135).

The nomogram showed good discrimination, with a C-statistic of 0.802 (95% CI, 0.772–0.832). The optimal cutoff based on the Youden index was a total score of 90, corresponding to a sensitivity of 75.0%, specificity of 72.5%, positive predictive value of 68.5%, and negative predictive value of 78.8%. The calibration curve showed good agreement between predicted and observed probabilities, and the Hosmer-Lemeshow test indicated acceptable calibration (*P* = 0.385). Decision curve analysis showed that the nomogram provided greater net benefit than treat-all or treat-none strategies across threshold probabilities of 15%–65%. After internal validation with 1,000 bootstrap resamples, the bias-corrected C-statistic was 0.789 and the calibration slope was 0.93, suggesting limited optimism (Figure 4). A schematic clinical stratification strategy is shown in Figure 5.

**Figure 4:**
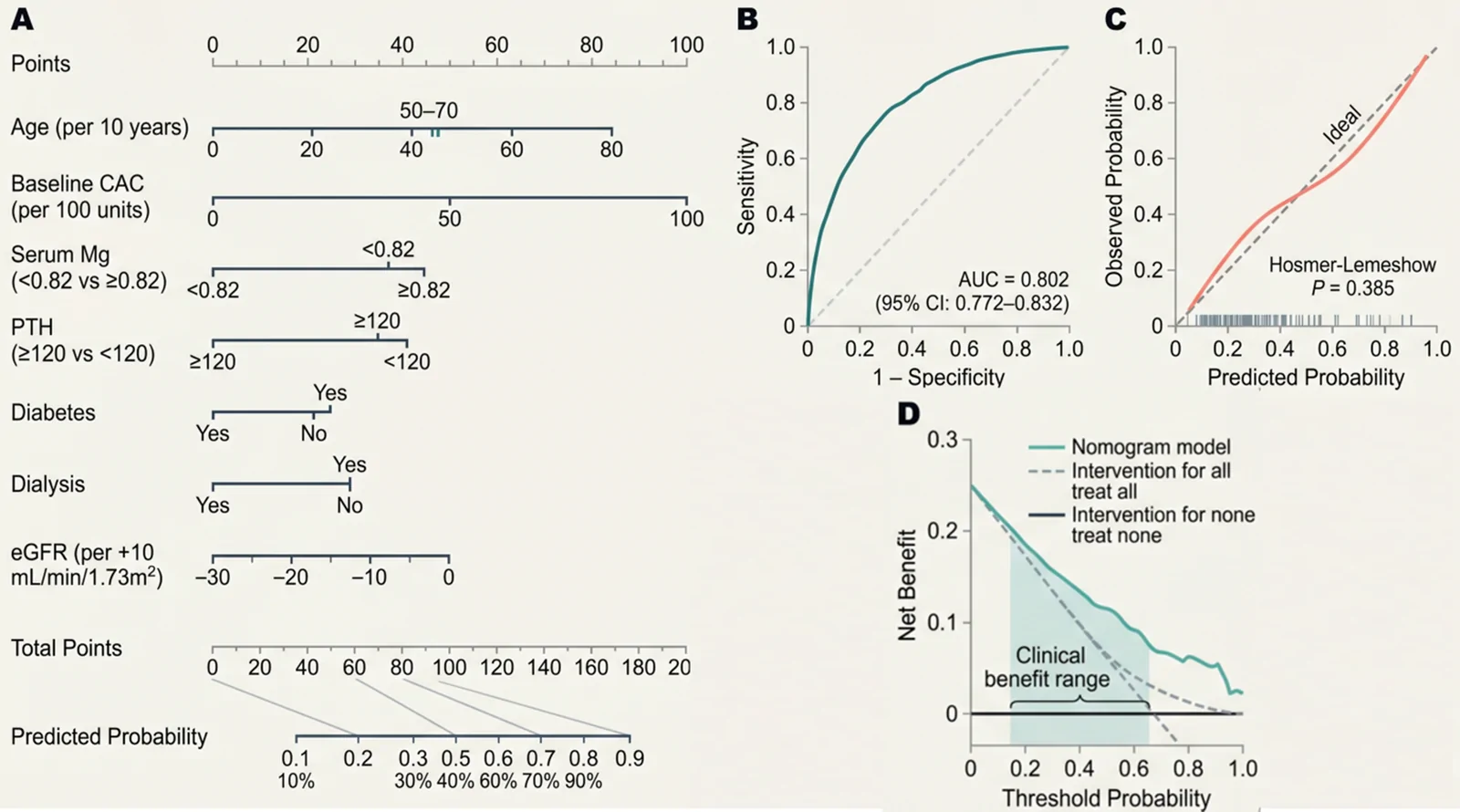
Risk prediction nomogram for rapid coronary artery calcification progression and model performance. **(A)** Nomogram predicting rapid CAC progression. **(B)** Receiver operating characteristic curve for the nomogram. **(C)** Calibration curve for the nomogram; the *x*-axis represents predicted probability and the *y*-axis represents observed probability. **(D)** Decision curve analysis for the nomogram. AUC, area under the curve; CAC, coronary artery calcification; DCA, decision curve analysis; eGFR, estimated glomerular filtration rate; PTH, parathyroid hormone; ROC, receiver operating characteristic.

**Figure 5:**
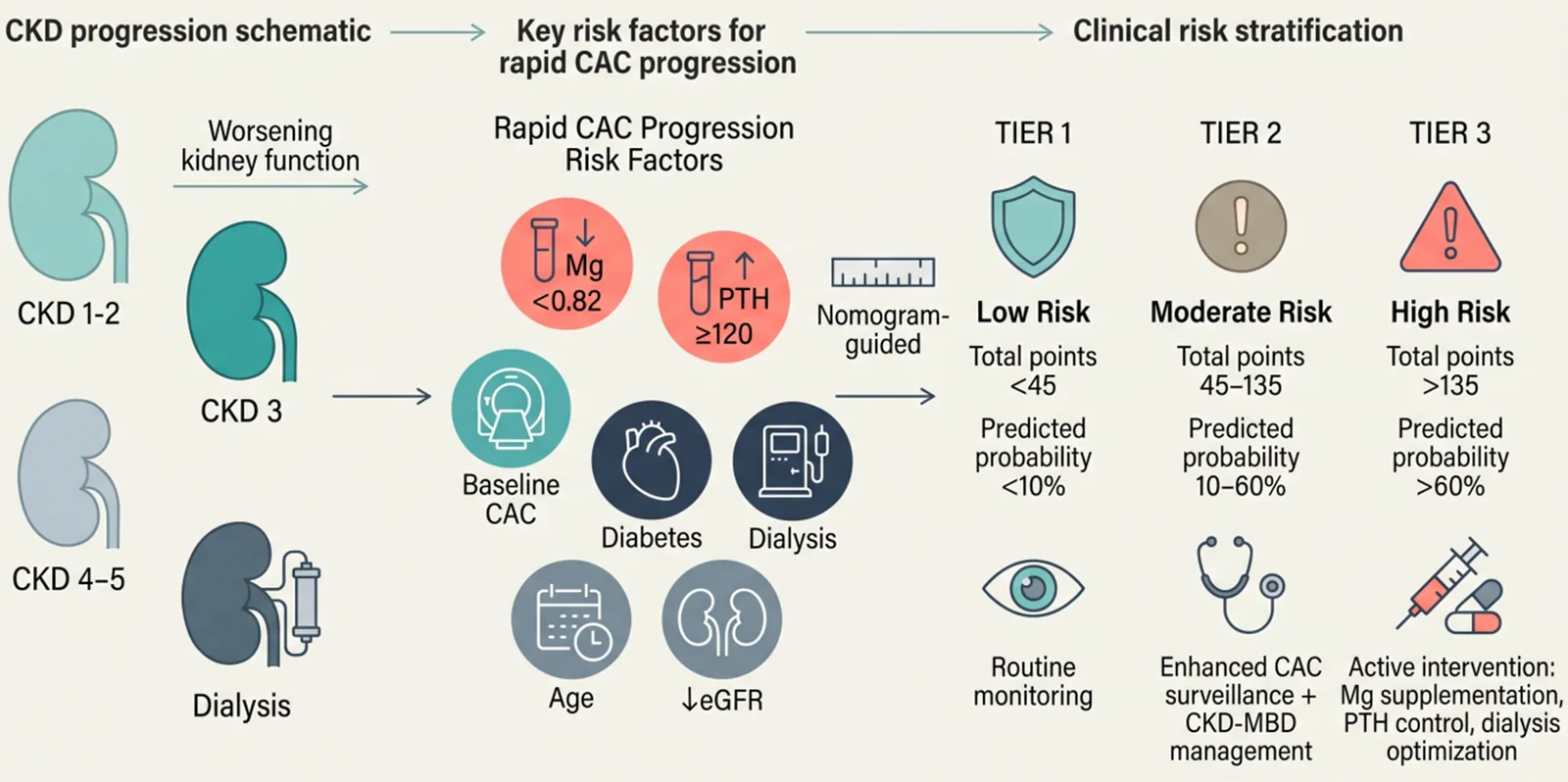
Key findings and clinical stratification strategy. The left panel illustrates CKD progression from stages 1-2 to stage 3, stages 4-5, and dialysis. The middle panel lists key risk markers for rapid CAC progression: serum magnesium <0.82 mmol/L, PTH ≥120 pg/mL, higher baseline CAC score, diabetes, dialysis status, older age, and lower eGFR. The right panel shows a stratified management concept based on nomogram score: low risk (<45 points; predicted probability <10%), medium risk (45-135 points; predicted probability 10%–60%), and high risk (>135 points; predicted probability >60%). CAC, coronary artery calcification; CKD, chronic kidney disease; CKD-MBD, chronic kidney disease-mineral and bone disorder; eGFR, estimated glomerular filtration rate; PTH, parathyroid hormone.

## Discussion

In this longitudinal cohort of 800 patients with CKD, CAC progression accelerated across worsening CKD stages and was fastest among dialysis patients. Serum magnesium and PTH showed nonlinear associations with rapid CAC progression, with clinically interpretable thresholds at 0.82 mmol/L and 120 pg/mL, respectively. A prediction model incorporating demographic, clinical, kidney function, and mineral metabolism variables showed good internal discrimination and calibration.

The annualized CAC progression rate in dialysis patients was approximately three times that observed in patients with CKD stages 1–2. This finding is consistent with prior studies and supports a close relationship between declining kidney function and accelerated vascular calcification (7, 8). A previous study comparing CAC progression across non-dialysis, hemodialysis, and peritoneal dialysis cohorts found faster annualized progression in dialysis patients than in non-dialysis patients, with no major differences between dialysis modalities, suggesting that the uremic milieu itself may be a major accelerator of calcification (14). A prospective study of 1,173 patients with CKD also found that severe CAC progression was associated with a higher risk of kidney failure requiring replacement therapy (15).

Biologically, declining kidney function impairs phosphorus excretion, and elevated serum phosphorus can induce osteogenic transformation of vascular smooth muscle cells (4). Even within the normal range, higher serum phosphorus has been associated with coronary artery, aortic, and valve calcification in older adults (16). In the present cohort, advanced CKD and dialysis patients had higher serum phosphorus levels and faster CAC progression. The marked progression observed in CKD stages 4–5 also suggests that vascular calcification may accelerate before dialysis initiation.

Although progression was slower in CKD stages 1–2, some patients in these stages still had continued CAC increases. This finding supports early cardiovascular risk assessment in CKD (2). The KNOW-CKD study similarly showed that CAC independently predicts cardiovascular events in non-dialysis CKD after adjustment for traditional risk factors (17). CAC assessment, when clinically appropriate, may therefore complement routine CKD risk management (3).

This study identified an L-shaped association between serum magnesium and rapid CAC progression. Below 0.82 mmol/L, lower magnesium was associated with substantially higher risk, whereas above this threshold the risk reduction plateaued. This finding suggests that maintaining adequate magnesium status may be relevant to calcification risk assessment, although interventional studies are needed before a treatment target can be recommended.

The anti-calcific effects of magnesium are supported by complementary physicochemical, cellular, and animal evidence. *In vitro*, magnesium inhibits extracellular hydroxyapatite formation and thereby reduces calcification of vascular smooth muscle cells (9). In human aortic vascular smooth muscle cells exposed to high phosphate, magnesium attenuates calcium deposition and preserves the expression and secretion profile of calcification-regulatory proteins, including matrix Gla protein (18). In experimental uremia, magnesium-containing treatment or dietary magnesium supplementation reduces vascular and soft-tissue calcification (19, 20). Additional cellular evidence indicates that magnesium can suppress BMP-2 and p65-NF-*κ*B signaling in TNF-α-treated endothelial cells (20). Collectively, these findings provide plausible physicochemical and cellular mechanisms for an anti-calcific effect of magnesium; however, their translation into clinical therapeutic benefit in CKD remains to be established. A recent clinical study suggests that markers of bone and mineral metabolism, including osteoprotegerin (OPG), may be associated with CAC progression (14). Beyond PTH, CKD-mineral and bone disorder-related factors such as phosphate, fibroblast growth factor 23, and sclerostin have also been investigated in relation to vascular calcification (31); however, the reported associations vary across populations and do not establish causality (21, 22). The role of OPG is particularly complex. Experimental evidence from OPG-deficient mice and recombinant OPG treatment suggests that OPG can inhibit arterial calcification (23, 24). In contrast, observational studies in hemodialysis and predialysis CKD cohorts have found that higher circulating OPG levels are associated with greater CAC burden or progression (14). This apparent paradox may indicate that increased circulating OPG is a compensatory marker of vascular injury and calcification rather than a causal promoter of these processes. As a natural calcium antagonist, magnesium may protect against calcification by modulating crystal formation and vascular smooth muscle cell phenotype. Evidence that statin therapy may not halt CAC progression in non-dialysis CKD further suggests that CKD-related calcification mechanisms differ from those in the general population and that CKD-mineral and bone disorder pathways require specific attention (25).

The overall prevalence of hypomagnesemia (<0.75 mmol/L) was 21.0% and increased to 26.0% among dialysis patients, consistent with previous literature (10). Dialysis patients may be prone to low magnesium because of dietary restriction, malabsorption, and dialysate magnesium concentration. The association between magnesium and CAC progression was slightly attenuated in dialysis patients (OR=1.89), possibly because magnesium values were less variable in this group or because stronger uremia-related drivers, including phosphorus and PTH, become more influential in advanced disease (26).

PTH showed a J-shaped association with rapid CAC progression, with a threshold of 120 pg/mL. Above this level, progression risk increased more steeply. A trend toward higher risk at very low PTH levels was also observed, although it was not statistically significant, and is consistent with reports of U-shaped associations in dialysis populations (12). In a study of 425 non-dialysis CKD patients, high PTH combined with severe baseline CAC was associated with a higher risk of major adverse cardiovascular events (27). Interactions among PTH, fibroblast growth factor 23, osteoprotegerin, sclerostin, and other bone-regulating molecules may contribute to the multifactorial nature of CAC in CKD (27).

Mechanistically, secondary hyperparathyroidism can promote high bone turnover, increase calcium and phosphorus release, raise the calcium-phosphorus product, and facilitate ectopic calcification (11). PTH may also act directly on vascular smooth muscle cells and promote osteogenic transition (4). The stronger association observed in CKD stages 4–5 and dialysis patients is consistent with the greater mineral metabolism disturbance in advanced kidney dysfunction (28).

Serum uric acid showed a linear positive association with rapid CAC progression (13), but it was not retained in the LASSO-derived prediction model. This suggests that its predictive contribution may be partly captured by stronger determinants such as eGFR and PTH. Uric acid may contribute to oxidative stress and inflammation, but its circulating level is also closely linked to kidney excretory function; adjustment for eGFR may therefore attenuate its independent predictive value.

The final nomogram used 7 readily available variables and showed good internal performance, with a bootstrap-corrected C-statistic of 0.789 and acceptable calibration. Its main practical value is that it integrates traditional risk factors with kidney function and CKD-mineral and bone disorder markers across a broad CKD population, including pre-dialysis and dialysis patients. Unlike prediction models focused on severe CAC in end-stage kidney disease (29), this model was developed across the full spectrum of CKD represented in the cohort. Emerging automated CAC scoring approaches using non-gated chest CT may further improve the feasibility of longitudinal CAC assessment in CKD cohorts (30). External validation is required before the model is used for clinical decision-making.

This study has limitations. First, this was a single-center retrospective cohort study and may have been affected by selection bias. Because inclusion required eligible serial chest CT examinations that had been obtained for clinical indications rather than through a universal screening protocol, the included patients may have had greater cardiovascular risk, a higher comorbidity burden, or more complex clinical conditions than the broader CKD population. This indication-based selection may limit the generalizability of our findings, and external validation in more representative CKD cohorts is warranted. Second, follow-up duration varied from 6 to 36 months, and shorter follow-up may affect estimation of long-term CAC trajectories. Third, magnesium, PTH, and uric acid were measured at baseline only, so time-varying effects could not be assessed. Fourth, definitions and cutoffs for rapid CAC progression vary across the literature, and consensus is still needed. Fifth, the exclusion of patients with recent adjustments to magnesium supplementation or phosphate-binder treatment may have selected a more treatment-stable cohort and may limit the generalizability of the findings. Moreover, information on medication changes during follow-up was incomplete, leaving the possibility of residual confounding by treatments affecting mineral metabolism. Although serum magnesium and PTH remained independently associated with rapid CAC progression after adjustment for serum calcium and phosphorus, the present analysis did not quantify their incremental predictive performance beyond these established mineral parameters. Moreover, the non-selection of calcium and phosphorus in the final LASSO model should be interpreted as model-specific variable selection rather than evidence that these factors are pathophysiologically unimportant or that magnesium and PTH have superior predictive value. Formal comparisons of nested models are required to determine whether magnesium and PTH meaningfully improve risk prediction beyond calcium and phosphorus. Finally, the nomogram requires validation in other ethnic groups and healthcare settings before broader implementation.

## Conclusion

CAC progression significantly accelerates as kidney function declines, peaking in dialysis patients. Low serum magnesium (<0.82 mmol/L) and elevated PTH (≥120 pg/mL) emerge as critical, nonlinear drivers of rapid calcification. Crucially, the 7-factor predictive nomogram developed in this study translates these complex longitudinal trajectories into a highly actionable clinical tool. By stratifying CKD patients into low-, moderate-, and high-risk tiers, this model empowers clinicians to move beyond generalized monitoring toward personalized cardiovascular management. For high-risk individuals, early identification via this nomogram can trigger targeted therapeutic interventions—such as stringent PTH control, personalized dialysis optimization, and potential magnesium supplementation—rather than merely intensifying surveillance. Ultimately, this predictive framework provides a practical pathway to improve cardiovascular outcomes in the vulnerable CKD population. Multicenter prospective studies are needed to validate this tool and evaluate the efficacy of these targeted interventions.

## Data Availability

The original contributions presented in the study are included in the article/Supplementary Material, further inquiries can be directed to the corresponding author.
