# Correction: Progression of coronary artery calcification in chronic kidney disease: nonlinear associations with serum magnesium and parathyroid hormone

**DOI:** 10.3389/fmed.2026.2003996

**Published:** 2026-09-29

**Authors:** Yutong Chen, Wenhao Qiu, Yinglan Shu, Lifeng Lu, Yindeng Luo

**Affiliations:** 1 Department of Radiology, The Second Affiliated Hospital of Chongqing Medical University, Chongqing, China; 2 School of Computer Science and Technology, Chongqing University of Posts and Telecommunications, Chongqing, China

**Keywords:** chronic kidney disease, coronary artery calcification (CAC), dose-response relationship, parathyroid hormone, progression trajectory, serum magnesium (Mg)

Affiliation 1 was erroneously given as “Department of Radiology, Second Affiliated Hospital of Chongqing Medical University, Chongqing, China”. The affiliation has now been updated to “Department of Radiology, The Second Affiliated Hospital of Chongqing Medical University, Chongqing, China”.

In the acknowledgements, the support statement was inadvertently omitted. The following sentence has now been added: “This work was supported by Key Laboratory of Intelligent Processing and Applications of Medical Imaging Big Data, Chongqing Municipal Health Commission and Engineering Research Center for Fundamental and Translational Intelligent Molecular Imaging, Chongqing Municipal Education Commission.” The correct acknowledgements statement is:

¨The authors thank the clinical and imaging staff of the Second Affiliated Hospital of Chongqing Medical University for their support in data collection. This work was supported by Key Laboratory of Intelligent Processing and Applications of Medical Imaging Big Data, Chongqing Municipal Health Commission and Engineering Research Center for Fundamental and Translational Intelligent Molecular Imaging, Chongqing Municipal Education Commission¨

The original version of this article has been updated.

